# Implementation of quality indicators for vulvar cancer in gynaecological cancer centres certified by the German Cancer Society (DKG)

**DOI:** 10.1007/s00432-024-05769-4

**Published:** 2024-05-10

**Authors:** Frederik A. Stuebs, Matthias W. Beckmann, Christian Dannecker, Markus Follmann, Monika Nothacker, Hans-Georg Schnürch, Linn Woelber, Simone Wesselmann

**Affiliations:** 1 Friedrich-Alexander-Universität Erlangen-Nürnberg, Department of Gynaecology and Obstetrics, Erlangen University Hospital, Comprehensive Cancer Centre Erlangen-European Metropolitan Area of Nuremberg (CCC ER-EMN), Universitaetsstrasse 21-23, 91054 Erlangen, Germany; 2 Department of Obstetrics and Gynaecology, University, Hospital Augsburg, 86156 Augsburg, Germany; 3 German Cancer Society e.V., 14057 Berlin, Germany; 4 AWMF-Institute for Medical Knowledge Management c/o Philipps-University Marburg, Karl-von-Frisch-Straße 1, 35043 Marburg, Germany; 5 Ehem. Frauenklinik Lukaskrankenhaus, Neuss, Germany; 6 Department of Gynaecology, University Medical Centre Hamburg-Eppendorf, Hamburg, Germany; 7 Dysplasia Centre Hamburg, Colposcopy Clinic at the Jerusalem Hospital, Hamburg, Germany

**Keywords:** Certified gynecological cancer centres, Vulvar cancer, Quality indicator, S2k-guideline

## Abstract

**Purpose:**

In 2018, the first guideline-based quality indicators (QI) for vulvar cancer were implemented in the data-sheets of certified gynaecological cancer centres. The certification process includes guideline-based QIs as a fundamental component. These indicators are specifically designed to evaluate the level of care provided within the centres. This article aims to give an overview of the developing process of guideline based-QIs for women with vulvar cancer and presents the QIs results from the certified gynaecological cancer centres.

**Methods:**

The QIs were derived in a standardized multiple step process during the update of the 2015 S2k guideline “Diagnosis, Therapy, and Follow-Up Care of Vulvar Cancer and its Precursors” (registry-number: no. 015/059) and are based on strong recommendations.

**Results:**

In total, there are eight guideline-based QIs for vulvar cancer. Four QIs are part of the certification process. In the treatment year 2021, 2.466 cases of vulvar cancer were treated in 177 centres. The target values in the centres for pathology reports on tumour resection and lymphadenectomy as well as sentinel lymph nodes have increased since the beginning of the certification process and have been above 90% over the past three treatment years (2019–2021).

**Discussion:**

QIs based on strong guideline recommendations, play a crucial role in measuring and allowing to quantify essential aspects of patient care. By utilizing QIs, centres are able to identify areas for process optimization and draw informed conclusions. Over the years the quality of treatment of vulvar cancer patients measured by the QIs was improved. The certification system is continuously reviewed to enhance patient care even further by using the outcomes from QIs revaluation.

## Introduction

Vulvar cancer (VC) is a rare malignant disease of the lower genital tract. The majority of vulvar cancer are vulvar squamous cell carcinoma (VSCC) (< 95%) (Diagnosis, Therapy, and Follow-Up Care of Vulvar Cancer and its Precursors 2015). The incidence of VC has been increasing over past years (Stuebs et al. 2020). The greatest increase in incidence was noticed in women below 70, but the highest incidence remains in women above 70 (median age: 73). In 2020, 3.090 women in Germany were diagnosed with VC and 973 died of the disease. Fortunately VC is diagnosed at an early stage in most cases (65–69%) and the relative 5 year overall survival for all stages is 70% (Krebs in Deutschland für 2019/2020). VC is caused by two known pathways for tumorigenesis: human papillomavirus-dependent pathway characterized by p16 overexpression like cervical cancer and a human papillomavirus-independent pathway linked to lichen sclerosus, characterized by TP53 mutation (Woelber et al. 2021; Beckmann et al. 2021a; Fehm et al. 2022). Early stages ofvulva cancers are treated by surgery in combination with adjuvant radiotherapy depending on the tumour stage (Zeitoun et al. 2022).

In Germany, gynaecological cancer centres are certified by the German Cancer Society (DKG) together with the Germany Society for Gynaecology and Obstetrics (Deutsche Gesellschaft für Gynäkologie und Geburtshilfe e. V. [DGGG]). The system was initiated in 2008 (Beckmann et al. 2014; Stuebs et al. 2023). In certified gynaecological centres, patients receive comprehensive care throughout their treatment pathway within an interdisciplinary and multi-professional network. In addition to certified gynaecological cancer centres gynaecologic dysplasia units and dysplasia consultations are obliged to offer diagnosis and treatment for (pre-)neoplastic lesions of the vulva in accordance with the guidelines (Schulmeyer et al. 2023). To obtain certification, all medical disciplines involved must demonstrate that they adhere to current German official guidelines and meet specific qualitative and quantitative standards. These standards are outlined in a catalogue of requirements and a data sheet. The quality indicators derived from these guidelines play a crucial role in maintaining these standards (Kowalski et al. 2017). The primary objective of the DKG certification system is to ensure a high level of quality in the treatment of cancer patients within certified gynaecological cancer centres (Stuebs et al. 2023; Rückher et al. 2022).

The first guideline for women with vulvar cancer was published in 2008 and updated in 2015. Because of the low incidence of VC and a lack of a sufficient number of randomized controlled trials (RCT), meta-analyses and systematic reviews, the current form of the guideline is consensus-based (S2K-Guideline). The German S2k-guidelines are based on a formal consensus-building process by a representative interdisciplinary and interprofessional expert team including patient representatives, covering relevant guideline topics (Becker et al. 2018). The guideline was led by the German Cancer Society (DKG), represented by the Working Group on Gynecological Oncology (AGO) and by the German Society of Gynecology and Obstetrics (DGGG), the latter funding guideline development. Methodological support and neutral moderation of consensus processes was provided by the Association of the Scientific Medical Societies in Germany (AWMF) (Diagnosis, Therapy, and Follow-Up Care of Vulvar Cancer and its Precursors 2015).

**Fig. 1:**
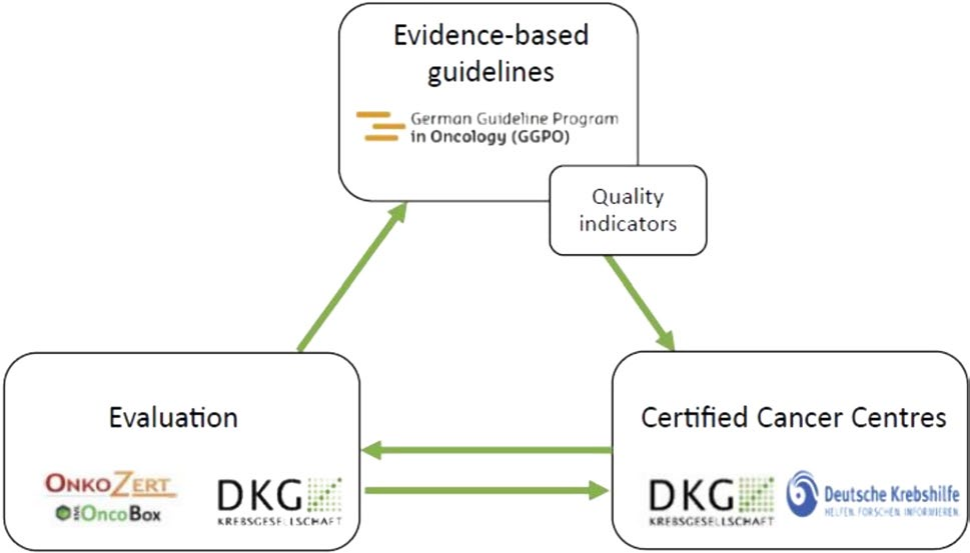
Quality cyclce in oncology (Rückher et al. 2022)

The German National Cancer Plan has led to the establishment of a quality cycle in oncology, which encompasses the interaction of guidelines and QIs development, their implementation in certified centres, and the utilization of outcomes for quality assurance and ongoing improvement of health care. The quality cycle serves as an interdisciplinary network, as depicted in Fig. 1 (Stuebs et al. 2023; Rückher et al. 2022). This article presents the methodology of QI development for vulvar cancer in the context of clinical guideline development and analyses the results of these QIs from the certified cancer centres (Rückher et al. 2022; Langendam et al. 2020; Nothacker et al. 2016).

Quality indicators are measurable elements; their collection serves to assess the quality of the underlying treatment structures, processes or results. QIs are an important tool in the management of quality. Their aim is to improve the quality of medical care by addressing areas with potential for improvement along the patient pathway, critically reflecting on and, if necessary, improving the results of care (Nothacker et al. 2011).

## Methods

Potential QIs for S2k-guideline vulvar cancer were selected by members of the working group Vulva and Vagina of the AGO. The focus was on important interdisciplinary interfaces (especially with pathologists) as well as on essential treatment indications (Diagnosis, Therapy, and Follow-Up Care of Vulvar Cancer and its Precursors 2015). The potential QIs were circulated and discussed among the working group members. Only strong recommendations of the guideline with a grade of recommendation “A” according an intervention “should/should not” (German: “soll/soll nicht”) are eligible to be selected as QI candidates since it could be expected that the implementation of these recommendations will have a positive impact on the outcome of the patients in the addressed patient group (German Guideline Program in Oncology 2021). Therefore the recommendations should be as specific as possible (German Guideline Program in Oncology 2021). These strong recommendations were translated in potential QIs. After approval by all members, the QIs were included into a preliminary version of a QI-chapter for the S2k guideline. This chapter was discussed in a telephone conference with one representative each of the DKG certification system, the Association of German Tumour Centres (ADT) and the German Guideline Program in Oncology (GGPO), in order to compare or establish the measurability with the currently existing documentation systems. An extern methodologist from the AWMF's subsequently assessed the results of the telephone conference. In the next step, the patient representatives examined the template, evaluated it and made suggestions. These were viewed by the methodologists. A set of final QIs was than generated by the working group members.

In the following section the set of vulvar cancer QIs and the results of the implemented QIs will be presented.

## Results

In 2014, members of the working group Vulva and Vagina of the AGO selected vulva cancer QIs on the basis of 49 strong recommendations of the S2k-guideline. In total, eight QIs were included in the final set of QIs (see Table 1). Of these QI, 5 were included in the data sheet for gynaecological cancer centres for the treatment year 2016: “Details given in the pathology report at first diagnosis and tumour resection” (QI1), “Details in the pathology report with lymphadenectomy” (QI2), “Local radical excision” (QI4), “Inguino-femoral staging” (QI6) and “Sentinel Lymph node Biopsy” (QI7). Consequently, the corresponding tumour-specific QIs also need to be documented (Kurzprotokoll zur Sitzung der Zertifizierungskommission Gynäkologische 2017). In 2017, the absolute patient numbers for the numerators and denominators of the QIs were reported for the first time and not only the median and range as in the previous annual report (Stuebs et al. 2023). In the treatment year 2019, the QI “Local radical excision” (QI4), was excluded from the data sheet because it combined two conditions in the numerator (R0 and local resection), and therefore was no longer reported (Kurzprotokoll zur Sitzung der Zertifizierungskommission Gynäkologische 2019).

**Table 1:** Quality indicators (QIs) for vulva carcinoma as defined in the S2k-guideline

QI		
1	Details given in the pathology report at first diagnosis and tumor resection	Numerator: no. of patients with pathology reports including details on: histological type (WHO)—grading—evidence/absence of lymphatic or venous invasion (L and V status)—evidence/absence of perineural sheath infiltration (Pn status)—staging (pTNM)—depth of invasion and extent in millimeters in pT1a1—three-dimensional tumor size in centimeters (starting from pT1b1)—metric measurement of the minimum distance of the carcinoma and the VIN to the vulvar resection margin in the histological specimen—If the vulvo-vaginal or vulvo-anal junction and, if applicable, the urethra have been resected, metric measurement of the minimum distance to the vulvo-vaginal or vulvo-anal or urethral resection edge—metric specification of the minimum distance to the soft tissue resection edge (basal edge)
		Denominator: all patients with a first diagnosis of vulvar carcinoma and tumor resection
2	Details in the pathology report with lymphadenectomy	Numerator: no. of patients with pathology reports including details on:—no. of affected lymph nodes relative to removed lymph node—correlation with site of biopsy removal (inguinal/pelvic)—details of the absence/presence of capsular infiltration by the lymph-node metastasis and/or detection of lymph vessel invasions in the perinodal fatty tissue and/or the lymph node capsule—details of the largest extent of the largest lymph-node metastasis
		Denominator: all patients with vulva carcinoma and lymphadenectomy
3	Pretherapeutic staging	Numerator: no. of patients with pretherapeutic reports including details on: depth of invasion—gynecological examination of the entire anogenitalarea including details on: determine the clinical seize of the tumor—determine the extent of tumor including the documentation of the extent of the tumor onto urethra, vagina, anus, bones (cT)—examination of the regional lymphatic drainage (palpation of the groin)
		Denominator: patients with a histologically comfirmed (Biopsy or exzision) first diagnosis vulva carcinoma
4	Local radical exzision	Numerator: no. of patients with local radial excision with R0 resection
		Denominator: no. of patients with a first diagnosis of vulvar cancer (T1a or T1b tumor)
5	Omission of the inguino-femoral staging	Numerator: no. of patients with an operative staging of the inguinofemoral lymph nodes
		Denominator: all patients with a first diagnosis of: vulvar cancer ≤ pTa1 or basal-cell carcinoma any clinical or pathological tumor stage or verrucous carcinoma any clinical or pathological tumor stage
6	Inguino-femoral staging	Numerator: no. of patients with operative staging of the inguino-femoral lymph nodes
		Denominator: all patients with first diagnosis of vulvar cancer ≥ pT1b not basal-cell carcinoma or verrucous carcinoma
7	Sentinel lymph node biopsy	Numerator: all patients with: tumor size < 4cm—unifocal tumor, no clinical sign of lymph node metastasis—pathological ultrastaging of the lymphnodes
		Denominator: all patients with first diagnosis of vulvar cancer and Sentinel lymph node biopsy
8	Psycho-oncology consultations	Numerator: no. of patients offered psycho-oncology consultations
		Denominator: all patients with first diagnosis of vulvar carcinoma tumor recurrence or metastasis

The initial certification of gynaecological cancer centres took place in 2008. Since then, the number of these centres has grown to 189 as of December 31, 2023 (Map 2024). In the treatment year 2021 in total 16.272 primary cases with the first diagnosis of a gynaecological cancer were treated in certified cancers. Vulvar cancer was the fourth most common primary cancer of the female genital tract (primary cases) [n = 1.705 (10.48%)] after endometrial cancer [n = 5.244 (32.23%)] ovarian cancer [n = 4.373 (26.87%)] and cervical cancer [n = 2.809 (17.26%)] (Kennzahlenauswertung 2023). The total number of primary cases treated in certified gynaecologic cancer centres has increased from the treatment year 2015 (n = 11.587) to 2021 (n = 16.272). Of the 2.466 patients with a first diagnosis of vulvar cancer in Germany, 1.705 were treated in centres. This represents 52.44% of the incident vulvar cancer cases in Germany.

Since 2019 (referring to the treatment year 2017), the results for five QIs (since 2021 for four QIs) are annually reported by the gynaecological cancer centres and published in the annual reports including median, range and numbers of included patients (Jahresbericht der deutschen Krebsgesellschaft (DKG) 2023).

### QI1: Details given in the pathology report at first diagnosis and tumour resection

This QI comprises details given in the pathology report for all patients with a first diagnosis of vulvar carcinoma and tumour resection. The number of centres meeting the target value (TV) of ≥ 80% has increased steadily over the years. For the treatment year 2021 96.57% of the centres met the TV.

### QI2: Details in the pathology report in the case of lymphadenectomy

This QI comprises details given in the pathology report for all patients with a diagnosis of vulvar carcinoma and lymphadenectomy. The number of centres meeting the TV has been 16.42% in the first year of reporting this QI. Since than the percentage of centres meeting the TV was above 98%. In the treatment year 2021, one centre did not meet the TV of ≥ 80%.

### QI4: Local radical excision

This QI comprises all patients with first diagnosis of vulvar cancer (T1a or T1b tumour) with local radial excision (R0). In the treatment year 2017, 28.99% of centres met the TV of at least 80% of complete excisions. In 2018, this value increased to 91.16%. In the following years this QI was no longer documented by the gynaecological cancer centres as described above.

### QI6: Inguino-femoral staging

Every women with a squamous vulvar cancer ≥ pT1b is supposed to get a surgical inguino-femoral staging. The numbers of centres meeting the TV of at least 90% for inguino-fermoral staging is between 60 to 70% for the treatment years 2018 until 2021.

### QI7: Sentinel lymph node biopsy

In 2017, 20.16% of centres met the TV, it increased to 86.96% in the treatment years 2018 and in the treatment year 2021 91.14% of the centres fulfilled the TV of 80% minimum.

## Discussion

The certification process is based on recommendations from interdisciplinary, formally consensus-based guidelines, mostly including also a formally evidence-based approach. We explained above the steps involved in developing QIs for the S2k-guideline Vulvar Cancer, including choice of guideline approach, selection and evaluation of the QI prior to their implementation in the certification process. QIs are utilized to evaluate the extent to which guideline recommendations are being followed in clinical practice. Their use is specifically possible for patients who are treated in certified oncological cancer centres due to favourable documentation (Stuebs et al. 2023).

**Fig. 2:**
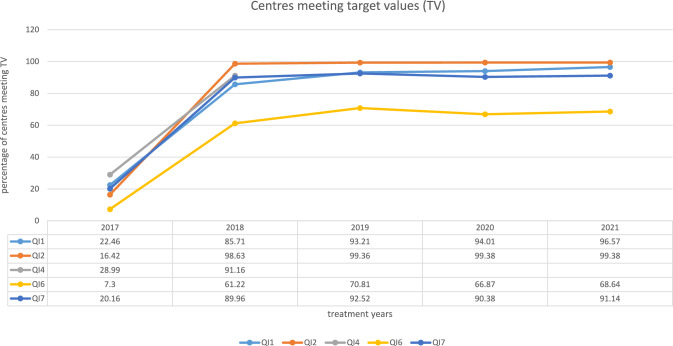
Centres meeting target values (TV) over time (years refer to the treatment years)

The five quality indicators of the guideline show very good results in the certified centres in the treatment years 2017–2021 resp. 2018 for QI4 (see Table 2 and Fig. 2).

**Table 2:** QIs over time (the years refer to the treatment years)

Quality indicator	2017			2018			2019			2020			2021		
	Results/Cases in total all sites	Median per site [range]	Centers meeting (TV)	Results/Cases	Median per site [range]	Centers meeting (TV)	Results/Cases	Median per site [range]	Centers meeting (TV)	Results/Cases	Median per site [range]	Centers meeting (TV)	Results/Cases	Median per site [range]	Centers meeting (TV)
1															
N	1025	6 [0–67]	22.46% (31/138)	1155	7 [0–62]	85.71% (126/147)	1332	6 [0–59]	93.21% (151/162)	1340	7 [0–44]	94.01% (157/167)	1367	6 [0–57]	96.57% (169/175)
D	1274	7 [1–68]		1329	7 [1–62]		1414	7 [1–60]		1418	7 [1–45]		1452	7 [1–57]	
2															
N	811	4 [0–63]	16.42% (22/134)	812	4 [1–46]	98.63% (144/146)	854	4 [1–52]	99.36% [155/156]	852	4 [1–35]	99.38% (160/161)	876	4 [1–54]	99.38% (159/160)
D	849	4 [1–63]		830	4 [1–46]		863	4 [1–52]		864	4 [1–35]		888	4 [1–54]	
4															
N	1060	6 [0–55]	28.99% (40/138)	1128	6 [1–53]	91.16% (134/147)	–	–	–	–	–	–	–	–	–
D	1140	6 [1–56]		1207	7 [1–55]		–	–	–	–	–	–	–	–	–
6															
N	876	5 [1–57]	7.30% (10/137)	943	5 [0–43]	61.22% [90/147]	999	5 [0–40]	70.81% (114/161)	989	5 [0–24]	66.87% (111/166)	1066	5 [0–49]	68.64% (116/169)
D	1003	6 [1–58]		1057	6 [1–45]		1101	5 [1–42]		1093	5 [1–25]+6		1172	5 [1–50]	
7															
N	559	3 [0–49]	20.16% (25/124)	599	3 [0–41]	86.96% (120/138)	687	4 [0–48]	92.52% (136/147)	680	3 [0–33]	90.38% (141/156)	717	3 [0–48]	91.14% (144/158)
D	665	4 [1–55]		675	4 [1–41]		739	4 [1–51]		733	3 [1–33]		778	3,5 [1–49]	
Total number of pt with vulva cancer treated in certified gynecological cancer centers	2.168		2269			2368			2370			2466			

*TV* target value

In the pathology reports for women with primary vulvar cancer the following details are included: histological type (WHO), grading, presence/absence of lymphatic or, venous invasion or perineural sheath infiltration (L, V or Pn-status), depth of invasion and extent in millimetres in pT1a1, three-dimensional tumour size in centimetres (starting from pT1b1), metric measurement of the minimum distance of the carcinoma and the VIN to the vulvar resection margin in the histological specimen. Especially the depth of invasion as described in the S2k-guideline is important for the surgical treatment of women with vulvar cancer (Wilkinson et al. 1982). Vulvar cancers with more than 1mm depth of invasion need a surgical staging of the groin nodes (Diagnosis, Therapy, and Follow-Up Care of Vulvar Cancer and its Precursors 2015). This QI has developed very well over the years and has steadily improved. The report of presence/absence of lymphatic, venous invasion and especially perineural sheath infiltration (L, V or Pn-status) was one of the main reasons for centres not to fulfil the TV in the first years. Special training for pathologist was therefore performed in the centres. Another common reason was discovered: the cancer had been completely removed in the biopsy and there was no tumour left in the surgical specimen so no detailed report was possible (Kennzahlenauswertung 2023).

For all patients with vulvar cancer and lymphadenectomy there should be a detailed pathology report including the following: number of affected lymph nodes relative to removed lymph nodes, correlation with site of biopsy removal (inguinal/pelvic), details of the absence/presence of capsular infiltration by the lymph-node metastasis and/or detection of lymph vessel invasions in the perinodal fatty tissue and/or the lymph node capsule. For the treatment years 2019–2021 all but one centre fulfilled the TV of 80% complete pathology reports. In 2019, details for absence/presence of capsular penetration by the lymph-node metastasis was missing in the report (Kennzahlenauswertung 2021). In 2020, one of the two patients treated in the centre did not agree for inguinal lymph node staging and in 2021, a pathologist not familiar with the QI was new in the pathology department. This member of staff has been trained for requirements of the pathology reports (Kennzahlenauswertung 2022, 2023). Currently there is no minimum number of vulvar cancer that has to be treated in one centre in order for the centre to be certified. Data on vulvar cancer about a positive association between a higher number of patients and improved quality is lacking, research on other malignancies indicate a positive impact of certification. For breast cancer a minimum number of 100 primary breast cancer patients per year is required for certification (Zentren-Brustkrebszentren 2024).

In 2018, the TV of at least 80% “local radical excisions” were reached in 91.16% of the centres. 13 out 147 centres had to justify R0 rates below 80%. In the audits, they often stated that an operation or a re-resection was declined by the patients (for example, due to advanced age) or that a complete vulvectomy, despite T1a/b or extensive vulvar intraepithelial neoplasia (VIN), was necessary (Kennzahlenjahr 2020). It was then decided by the certification commission to exclude this QI from the data sheet because it combined two conditions in the numerator (R0 and local resection) (Kurzprotokoll zur Sitzung der Zertifizierungskommission Gynäkologische 2019).

The TV of ≥ 90% of “Inguino-femoral staging” in patients with vulvar carcinoma ≥ pT1b (QI6) were fulfilled by 61.22% and 70.81% of the centres in the years 2018–2021. The centres that did not reach the target value were not the same every year. As reasons for not reaching the target value, age of the patient in connection with dementia and multimorbidity are almost consistently mentioned. In addition, palliative radiation instead of surgery, concomitant other cancer diseases, and the patient's rejection of lymph node staging also play a role (Kennzahlenauswertung 2023). The reasons mentioned for not reaching the TV could be plausibly explained in the audits by the centres. The large variability in values is due to the sometimes-small number of patients in the denominator, which often resulted in only 1 case being enough falling below the target value (Kennzahlenauswertung 2022).

The QI7 “Sentinel Lymph Node Biopsy” comprises all women with first diagnosis of vulvar cancer < 4 cm, unifocal and no sign of lymph node metastasis. A pathological ultrastaging of the lymph nodes has to be provided, if routine processing (HE) does not show any metastasis (Diagnosis, Therapy, and Follow-Up Care of Vulvar Cancer and its Precursors 2015). In 2018, 86.96% of centres met the TV’s and in the following years the centres meeting the TV’s were above 90%. In 2021, 14 centres (9%) did not meet the target requirement. One centre stated that they have previously carried out a complete examination of the lymph nodes with microscopic examination of the entire material, but ultrastaging has now been implemented. Common reasons for not meeting the target requirement were discrepancies between clinical and postoperative tumour size, absence of clinical tumour size, and patient refusal. The experts reviewed the cases in the audit (Kennzahlenauswertung 2023). The centres responded with trainings, establishing standard operating procedures (SOP) and raising awareness among their employees (Kennzahlenauswertung 2022).

There are limitations in the process of establishing and implementing Quality Indicators (QIs) in data sheets for certified centres. The consideration of patients' quality of life is not taken into account when setting up QIs. It is not possible to cover the entire content of a guideline with QIs. QIs are evaluated and discussed during onsite audits one year after the treatment year of the patients, which means that the structure and healthcare process may has already varied in the local centre, resulting in different framework conditions for the QI results. The introduction of digitalization in hospitals could be an opportunity, as measuring and reflecting on QIs alongside patient treatment would allow for timely responses to deviations from the quality objectives. The acceptance of clinicians is crucial for the successful implementation of QIs, and the practicality of documenting the QIs needs to be considered. Taking these factors into consideration, only a small subset of QIs is transferred to data sheets. When a guideline is published the underlying data can be outdated due to the time-consuming process of establishing the guideline. While treating women with vulvar cancer the clinicians also need to consider current international recommendations and guidelines (Abu-Rustum et al. 2024; Oonk et al. 2023).

Using guideline based QIs to implement and assess medical care for women with vulvar cancer can have a positive impact on measuring of high-quality patient care, and this aids in the improvement of quality in diagnosis and treatment over the long term (Rückher et al. 2022; Butea-Bocu et al. 2021; Beckmann et al. 2011; Haj et al. 2017; Kreienberg et al. 2018; Trautmann et al. 2018).

Furthermore, the outcomes of the QIs can be utilized during the certification procedure to pinpoint regions where enhancements can be made. In the event that centres do not meet the TV, they have the chance to explain the deviation and hold discussions about it during audits. At this point, appropriate actions can be agreed upon by the centre and the auditors, which are aimed at improving the QIs results (Rückher et al. 2022). In the subsequent year’s audit, the review of these measures allows for an evaluation of their effectiveness. As a result, the certified centres establish a consistent and successful quality improvement process that aligns with guideline QIs. Results of QIs may also be useful in the update process of guidelines as they help to evaluate current medical practice.

Implementation and evaluation of systematic quality improvement measures can be particularly beneficial for less prevalent cancer types like vulvar cancer. This approach helps generating broader databases, expands knowledge and is expected to improve care and treatment for affected women as demonstrated for other gynecologic cancers such as cervix cancer, endometrial cancer or ovarian cancer in WiZen-Trial (Schmitt et al. 2023).

## Declarations

### Data availability statement

The datasets generated during and/or analysed during the current study are available from the corresponding author on reasonable request.
